# Appropriate Development of the Liver Treg Compartment Is Modulated by the Microbiota and Requires TGF-*β* and MyD88

**DOI:** 10.1155/2014/279736

**Published:** 2014-08-07

**Authors:** Ann Maria, Kathryn A. English, James D. Gorham

**Affiliations:** ^1^The Geisel School of Medicine at Dartmouth, Department of Microbiology and Immunology, DHMC, One Medical Center Drive, Lebanon, NH 03756, USA; ^2^The Geisel School of Medicine at Dartmouth, Department of Pathology, DHMC, One Medical Center Drive, Lebanon, NH 03756, USA

## Abstract

Neither the early postnatal development of the liver Treg compartment nor the factors that regulate its development has been characterized. We compared the early developmental patterns of Treg cell accumulation in murine liver, thymus, and spleen. A FoxP3^EGFP^ reporter mouse was employed to identify Treg cells. Mononuclear cells were isolated from organs postnatally, stained for CD4, and examined by flow cytometry to enumerate FoxP3^+^CD4^hi^ cells. To assess roles for TGF-*β*1, MyD88, and TLR2, gene-specific knockout pups were generated from heterozygous breeders. To test the role of commensal bacteria, pregnant dams were administered antibiotics during gestation and after parturition. The pattern of appearance of Treg cells differed in liver, spleen, and thymus. Notably, at 1-2 weeks, the frequency of CD4^hi^ FoxP3^+^ T cells in liver exceeded that in spleen by 1.5- to 2-fold. The relative increase in liver Treg frequency was transient and was dependent upon TGF-*β*1 and MyD88, but not TLR2, and was abrogated by antibiotic treatment. A relative increase in liver Treg frequency occurs approximately 1-2 weeks after parturition that appears to be driven by colonization of the intestine with commensal bacteria and is mediated by a pathway that requires TGF-*β*1 and MyD88, but not TLR2.

## 1. Introduction

The immune system is tightly regulated, maintaining, in normal physiological conditions, a balance between immunity to challenge by pathogens and tolerance in order to suppress inappropriate immune responses. Regulatory T cells (Treg) have been recognized to play a major role in immune homeostasis by maintaining self-tolerance and preventing autoimmunity. Expression of the transcription factor forkhead box P3 (FoxP3) specifies the Treg lineage and confers suppressive function, through activation of a transcriptional program required for regulation [1–3]. The cytokine transforming growth factor beta1 (TGF-*β*1) plays a critical role in Treg development and function [4].

The liver can be considered an immune-privileged site [5] similar to other immune-privileged organs such as the eye and the gonads. The tolerogenic status of the liver is necessary because the liver receives blood not only from the systemic circulation via the hepatic artery, but also from the gastrointestinal (GI) tract via the portal vein. Portal vein flow results in a high concentration of non-pathogen-associated antigens reaching the liver, such as food antigens and bacterial breakdown products from commensal organisms residing in the gut. For example, lipopolysaccharide (LPS) is present in the portal venous blood at a concentration of about 1 ng/mL [6], a concentration which, if present in the systemic circulation, would lead to septic shock. To handle this immense load of bacteria-derived substances, the liver maintains a state of local immune tolerance, using a variety of mechanisms [5, 7]. The liver Treg compartment is one important component of the network of cells that mediates liver tolerance [8, 9].

Toll like receptors (TLRs) are pathogen recognition receptors that recognize conserved molecules found across diverse bacterial species; for example, TLR2 recognizes lipoteichoic acid present in most Gram-positive bacteria, whereas TLR4 recognizes LPS, present in most Gram-negative bacteria [10, 11]. TLRs play critical roles in linking the innate and adaptive branches of the immune system. TLR ligands induce dendritic cell (DC) maturation from an immature phenotype and upregulate MHC class II and costimulatory molecules, necessary for proper activation of T cells by DCs [12]. Myeloid differentiation primary response 88 (MyD88) is a universal cytosolic adaptor protein that is downstream of all bacterial-responsive TLRs.

While published studies have looked at the development of Tregs in the neonatal thymus [13], and spleen [14], the development of Tregs in the neonatal liver has not been previously studied. Here, we assess the development of the Treg compartment in the postnatal liver and examine the contributions of TGF-*β*1 and MyD88 to liver Treg development.

## 2. Materials and Methods

### 2.1. Mice

FoxP3^EGFP^ mice on the BALB/c background and TLR2 knockout mice were obtained from the Jackson Laboratory (Bar Harbor, ME). FoxP3^EGFP^.*Tgfb1* ^−/−^ knockout mice on the BALB/c background were generated by breeding FoxP3^EGFP^ with* Tgfb1* ^+/−^ heterozygous mice [15]. FoxP3^EGFP^ mice harbor a bicistronic* FoxP3* locus that coexpresses eGFP and FoxP3 [16]. The use of these reporter mice ensures that native FoxP3 protein remains unmodified, as it has been shown recently that a commonly used reporter mouse expressing a FoxP3^gfp^ fusion protein is a hypomorph with an abnormal Treg phenotype [17]. MyD88 knockout mice [18] were a kind gift from Dr. Brent Berwin at the Geisel School of Medicine. Timed pregnant C57BL/6 mice were obtained from the National Cancer Institute (NCI). Mice were bred at the Geisel School of Medicine according to Association for Assessment and Accreditation of Laboratory Animal care practices. FoxP3^EGFP^ and TLR2 knockout mice were genotyped by PCR as per the vendors' protocol. TGF-*β*1 knockout mice and MyD88 knockout mice were genotyped as previously described [19, 20], whereas Leadbetter et al. [20] indicate that PCR products expected from* Myd88* ^+/+^ mice and* Myd88* ^−/−^ mice are approximately 550 and 750 base pair (bp), respectively; in our hands these PCR products were approximately 600 and 300 bp, respectively. The mouse strain(s) (C57Bl/6 or BALB/c) used in each figure is indicated in the figure legends.

### 2.2. Isolation of Mononuclear Cells from Organs

Cardiac perfusion was carried out before removal and weighing of organs. Liver tissue was dissociated by chopping using a razor blade or by use of a tissue dissociator as per manufacturer's protocol (gentleMacs dissociator, Miltenyi Biotec) and subjected to treatment with 5 *μ*g DNAse and 500 Units of Collagenase (Sigma). A cell suspension of liver nonparenchymal cells (NPC) was obtained by filtering and removal of hepatocytes, followed by red blood cell (RBC) lysis. For spleen and thymus, cell suspensions were prepared by grinding between frosted glass slides, followed by filtering and RBC lysis.

### 2.3. Flow Cytometry

Prior to staining with specific antibody, nonspecific binding was blocked using Fc block (anti-mouse CD16/CD32, clone 93, eBioscience). Antibodies used for staining were anti-CD4 (clone GK1.5, eBioscience), anti-CD3 (clone 145-2C11, BD Pharmingen), and anti-FoxP3 (clone FJK-16s, eBioscience). Intracellular staining for FoxP3 was carried out using a kit according to the manufacturer's protocol (eBioscience). Cell staining was acquired on either FACS Calibur or BD Accuri C6. Flow data analysis was carried out using either FlowJo version 7.6.5 (TreeStar) software or BD Accuri C6 software (Version 1.0.202.1).

### 2.4. Antibiotic Treatment

Timed pregnant mice were treated beginning at about two weeks of gestation with the following antibiotics (in the drinking water): ampicillin (1 g/L), neomycin sulfate (1 g/L), metronidazole (1 g/L), and vancomycin (0.5 g/L). Sucrose (10 g/L) was also added to the water. Antibiotic treatment in the drinking water was maintained until pups were 8-9 days old. Antibiotic water was changed twice weekly.

### 2.5. Statistics

Significance was determined using either the nonparametric Mann Whitney *U*-test for two-group comparisons or a two-way ANOVA for kinetic data. Statistical analyses were carried out using GraphPad Prism software (Version 6.0). Significance is denoted as follows: ns not significant (*P* > 0.05), *P ≤ 0.05, **P ≤ 0.01, ***P ≤ 0.001, ****P ≤ 0.0001, or as indicated in the figures. Error bars indicate mean ± standard deviation. Box and whisker plots show 5th to 95th percentile.

## 3. Results

### 3.1. Postnatal Development of FoxP3^+^CD4^hi^ T Cells in Thymus, Spleen, and Liver

**Figure 1:**
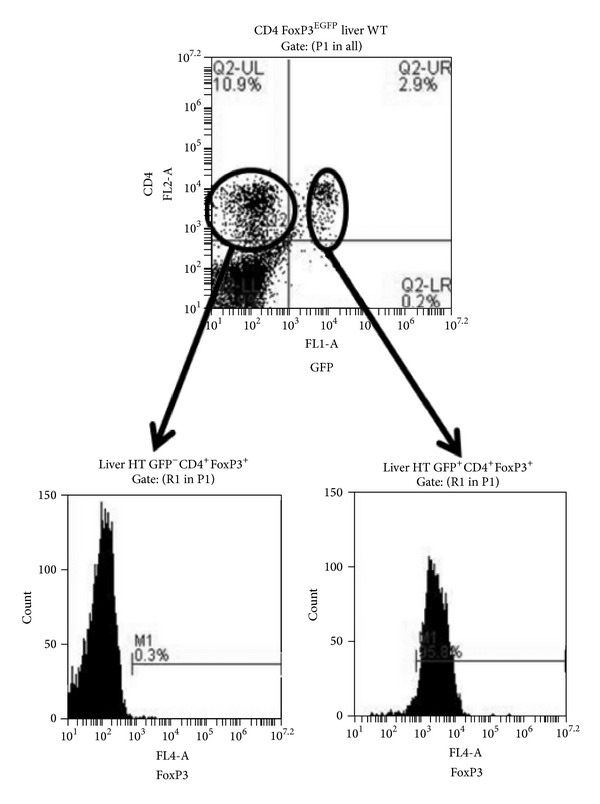
GFP expression is a reliable marker of FoxP3 expression in liver CD4^+^ cells from FoxP3^EGFP^ transgenic reporter mice. NPC were isolated from liver of adult BALB/c-background FoxP3^EGFP^ mice and CD4^+^ cells were sorted by GFP expression. Sorted cell populations were then analyzed by intracellular staining for FoxP3 expression.

We employed a reporter mouse that provides convenience in the detection of FoxP3 expressing cells [16]. In FoxP3^EGFP^ transgenic reporter mice, FoxP3 expressing cells are identified as eGFP^+^ and are readily detected on flow cytometry without needing additional manipulations, such as intracellular staining. It has been shown recently that transgenic mice in which the eGFP reporter is expressed as a fusion protein with FoxP3 have subtle immunologic abnormalities, owing to unexpected effects of the eGFP fusion partner on FoxP3 functionality [17]. For this reason, we used reporter mice in which the FoxP3 protein is expressed from a bicistronic reporter construct that coexpresses FoxP3 and eGFP as separate proteins [16]. We obtained evidence that the expression of GFP is a reliable marker for FoxP3 expression. Using cell sorting to isolate GFP^+^ and GFP^−^ populations, we analyzed sorted populations by intracellular staining for the nuclear protein FoxP3 (Figure 1). The GFP^−^ population did not show expression of FoxP3, whereas greater than 95% of the GFP^+^ population was positive for the expression of FoxP3.

Next, we assessed the development of FoxP3^+^CD4^hi^ T cells (regulatory T cells (Tregs)) in early postnatal thymus, liver, and spleen of FoxP3^EGFP^ reporter mice. We assessed both the frequency of Tregs, defined as the percentage of Tregs (GFP^+^ or FoxP3^+^) among CD4^hi^ T cells, and the density of Tregs in each organ, defined as Treg cells/mg.

**Figure 2:**
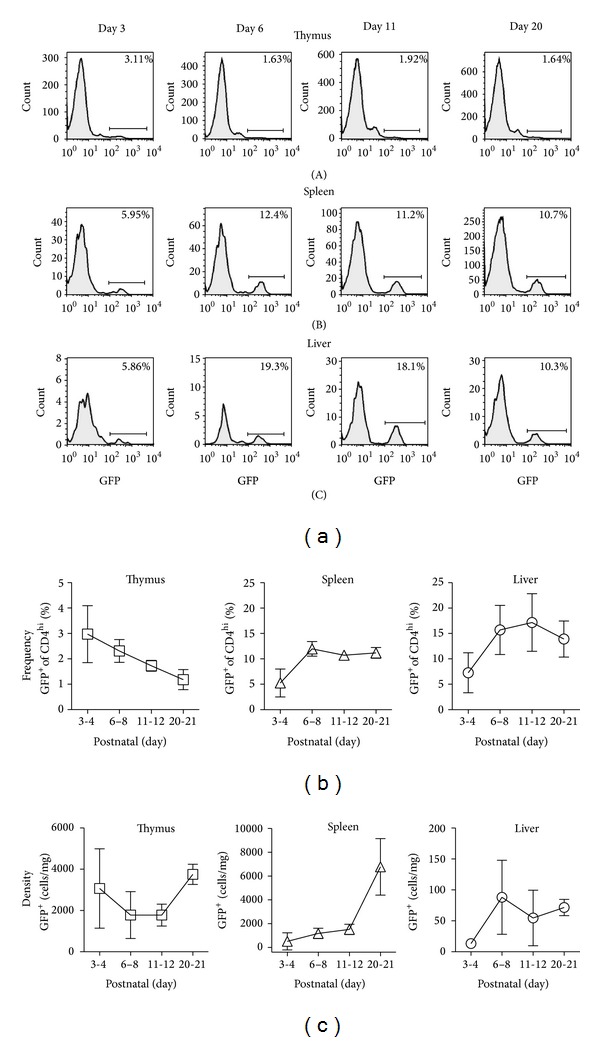
Postnatal development of FoxP3^+^CD4^+^ cells in thymus, spleen, and liver. NPC were isolated from thymus, spleen, and liver of BALB/c-background FoxP3^EGFP^ transgenic reporter mice of the indicated ages. (a) Individual GFP expression profiles of CD4^+^ T cells are shown. (b) Composite data from several mice are shown. Frequency indicates the percentage of CD4^+^ T cells that coexpress eGFP as a reporter of FoxP3 expression. *N* = 3 to 8 mice per time point. (c) Composite data from several mice are shown. Density indicates the number of CD4^+^eGFP^+^ cells per wet weight of the organ. *N* = 3 to 8 mice per time point.

Previous studies show that Tregs are detectable in thymus at postnatal days 3-4 [13, 21]. Consistent with this, about 3% of CD4^hi^ cells in FoxP3^EGFP^ mouse thymus were GFP^+^ at postnatal days 3-4 (Figures 2(a) and 2(b)). Over the next two to three weeks, the frequency of GFP^+^ cells among CD4^hi^ cells progressively decreased to ~1% by weaning age (day 20-21). The density of Tregs in thymus remained relatively constant throughout the neonatal period (range 1,200 to 4,000 cells/mg; Figure 2(c)). In this analysis, we did not specifically stain for CD8 and therefore cannot formally determine whether some of these cells represent CD4CD8 double positive (DP) cells. However, this percentage is likely to be quite small, as it has been shown in several previous studies that, in the postnatal period through adulthood, greater than 95% of CD4^+^FoxP3^+^ thymocytes are CD4^hi^CD8^neg^ single positive cells and less than 5% of CD4^+^FoxP3^+^ thymocytes are DP cells [13, 22].

In spleen, the frequency of Tregs was ~6% at days 3-4 and quickly increased, reaching a steady state of ~10–12% at days 6–8, a frequency maintained at 11-12 days and 20-21 days (Figures 2(a) and 2(b)). The density of Tregs in the spleen was 500 cells/mg at days 3-4 and progressively increased over the next several weeks. By weaning age, splenic Treg density increased significantly, to ~6,500 cells/mg (Figure 2(c)).

In liver, Tregs were detectable as early as days 3-4, with a frequency (~6%) comparable to that of spleen at similar age. At days 6–8 and 11-12, approximately 15–17% of CD4^hi^ T cells were Tregs. At days 20-21, the frequency was lower (13%; Figures 2(a) and 2(b)). At all ages tested, the density of Tregs in liver was lower than in either spleen or thymus (Figure 2(c)), consistent with the lower numbers of immune cells in this nonlymphoid organ.

**Figure 3:**
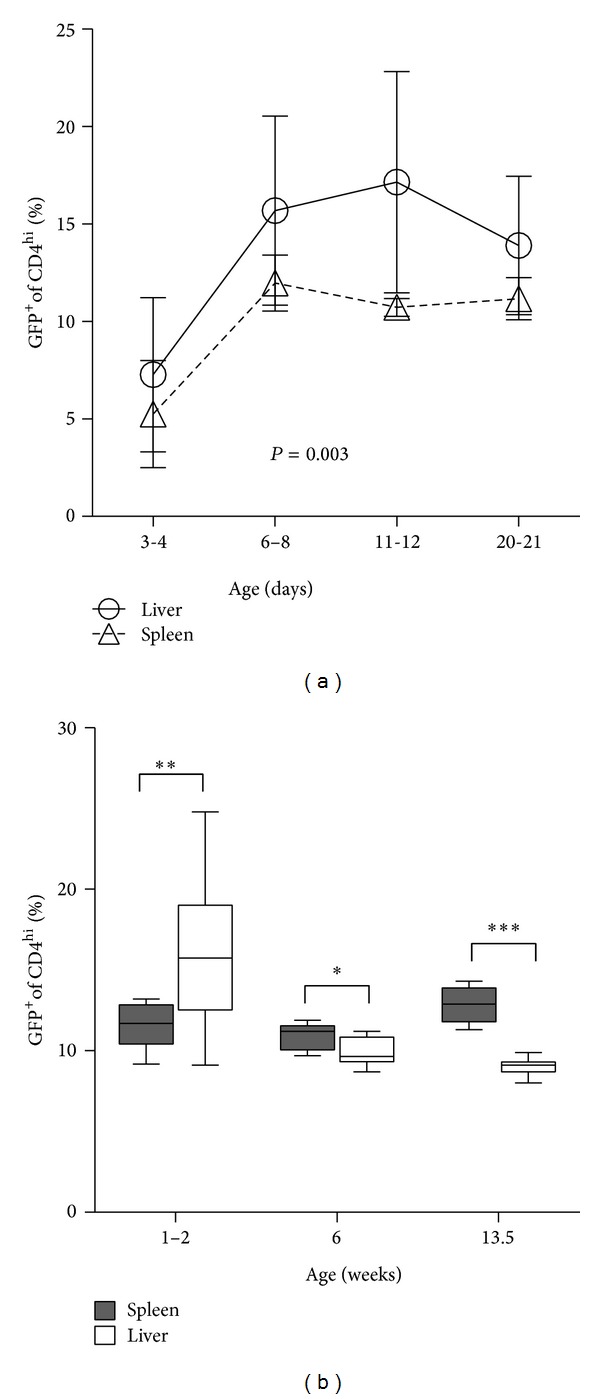
The increased frequency of FoxP3^+^CD4^+^ cells in postnatal liver reverses in the adult. (a) Liver and spleen FoxP3^+^CD4^+^ frequency data from BALB/c-background FoxP3^EGFP^ mice prior to weaning are displayed. Statistical analysis was by 2-way ANOVA. (b) FoxP3^+^CD4^+^ frequency data from preweaned mice (age 1-2 weeks) and adult mice (age 6 weeks; age 13.5 weeks) are shown. For 1-2 weeks, data for 6- to 8-day-old mice were combined with data from 11- to 12-day-old mice. *N* = 7 to 12 mice per time point. Statistical analyses used the nonparametric Mann-Whitney test.

A direct comparison of Treg frequency in the two organs shows that the spleen and liver were comparable at days 3-4 and 20-21. However, in between these two time points, the liver exhibited a significantly greater (~1.5-fold) Treg frequency (Figure 3(a)). Statistical analysis using ANOVA revealed that these curves are significantly different (*P* = 0.003). Assessing later time points, at 6 weeks, the relative frequency of Tregs in liver was lower than in spleen, a difference that was even more pronounced at 13.5 weeks (Figure 3(b)). Thus, at 1-2 weeks of age, the liver exhibits a relatively greater Treg frequency as compared with spleen; in the mature adult, the relative frequency is reversed, with liver showing lower Treg frequency among CD4^hi^ T cells.

### 3.2. TGF-*β*1 Is Required for the Normal Pattern of Development of FoxP3^+^CD4^hi^ T Cells in Thymus, Spleen, and Liver

Because TGF-*β*1 plays an important role in the ontogeny and function of Tregs, we analyzed its contribution to the early development of Tregs in thymus, spleen, and liver. To facilitate this analysis, we crossed BALB/c background FoxP3^EGFP^ mice with BALB/c background TGF-*β*1 knockout (*Tgfb1* ^−/−^) mice [15]. BALB/c* Tgfb1* ^−/−^ mice develop histologically and biochemically detectable necroinflammatory disease in liver and other organs beginning at around 10 days of age, caused by an influx of CD4^+^ T cells [23], and die at 15–17 days of age. Owing to this lethality, we could not measure Tregs at 20-21 days, so our analyses here are restricted to the 3-4, 6–8, and 11-12 day time points only. FoxP3^EGFP^ littermates with one intact* Tgfb1* allele (heterozygous* Tgfb1* ^+/−^ mice) were used as controls;* Tgfb1* ^+/−^ mice are healthy and phenotypically indistinguishable from wild type (*Tgfb1* ^+/+^) mice [19].

**Figure 4:**
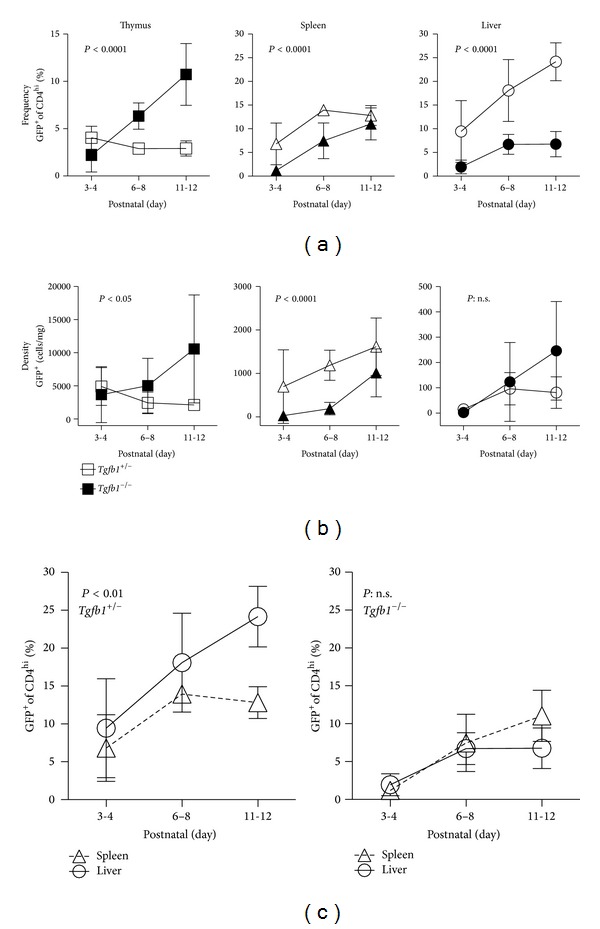
The increased frequency of FoxP3^+^CD4^+^ cells in postnatal liver depends on TGF-*β*1. NPC were isolated from thymus, spleen, and liver of BALB/c-background FoxP3^EGFP^.*Tgfb1* ^+/−^ mice and littermate FoxP3^EGFP^.*Tgfb1* ^−/−^ mice at the indicated ages. (a) Frequency data for FoxP3^+^CD4^+^ cells are shown. (b) Density data for FoxP3^+^CD4^+^ cells are shown. (c) Data for spleen and liver are shown for* Tgfb1* ^+/−^ mice and* Tgfb1* ^−/−^ mice. *N* = 4 to 9 mice per group at each time point. Statistical analyses were by 2-way ANOVA.

In thymus, Treg frequency in control* Tgfb1* ^+/−^ mice was ~4% at days 3-4 and diminished moderately over the next week (Figure 4(a)), indicating that the control mice exhibited the expected WT FoxP3^EGFP^ mouse pattern (Figure 2). In* Tgfb1* ^−/−^ mice at days 3-4, thymic Treg frequency was lower than in littermate* Tgfb1* ^+/−^ mice. The Treg frequency in* Tgfb1* ^−/−^ thymus increased dramatically over the next week, reaching 10% at days 11-12 (Figure 4(a)). The density of Tregs also increased in* Tgfb1* ^−/−^ thymus over this time period (Figure 4(b)). The dramatic increase in thymic Treg production at days 6–8 and 11-12 is consistent with previous reports showing a similar pattern in mice in which T cells have been rendered conditionally deficient in one of the key components of the TGF-*β* receptor [21, 24].

In spleen, Treg frequency in control* Tgfb1* ^+/−^ mice exhibited the expected WT FoxP3^EGFP^ mouse pattern; that is, Treg frequency was ~7% at days 3-4 and increased to a steady state of ~12 to 13% over the next week.* Tgfb1* ^−/−^ mice exhibited a significant delay in the development of Tregs in the neonatal spleen; at days 3-4 and 6–8, both frequency and density of Tregs in* Tgfb1* ^−/−^ spleen were significantly lower compared to* Tgfb1* ^+/−^ littermates. By days 11-12, there was no difference in either frequency or density between* Tgfb1* ^−/−^ spleens and* Tgfb1* ^+/−^ spleens (Figures 4(a) and 4(b)).

In liver, Treg frequency in control* Tgfb1* ^+/−^ mice was ~10% at days 3-4 and increased to over 20% over the next week, an increase similar to that observed in WT FoxP3^EGFP^ liver. As in spleen,* Tgfb1* ^−/−^ liver exhibited a significant delay in the development of Tregs; at days 3-4, the frequency of Tregs in* Tgfb1* ^−/−^ liver was significantly lower compared to littermate control* Tgfb1* ^+/−^ livers. At days 11-12, the frequency of Tregs among CD4^+^ T cells in* Tgfb1* ^−/−^ liver remained low (Figure 4(a)). This is a different pattern from that observed in* Tgfb1* ^−/−^ spleen, where the frequency of Tregs normalized by days 11-12. The low Treg frequency at day 11-12 is likely a function of the massive influx of (non-Treg) effector CD4^+^ T cells into liver (but not spleen) that occurs just prior to 11 days of age in* Tgfb1* ^−/−^ mice [23]. Indeed, at days 11-12, the absolute density of* Tgfb1* ^−/−^ liver Tregs surpassed that of littermate control* Tgfb1* ^+/−^ liver Tregs, consistent with a large overall T cell influx (Figure 4(b)).

Next, we examined these data to test the hypothesis that TGF-*β*1 is required for the transient increase in liver Tregs observed at 1-2 weeks of age. We compared the frequency of Tregs in liver with the frequency of Tregs in spleen in* Tgfb1* ^−/−^ mice, as well as in littermate control* Tgfb1* ^+/−^ mice. At days 11-12, littermate control* Tgfb1* ^+/−^ mice had a significantly higher frequency of Tregs in liver as compared with spleen (*P* < 0.01 by ANOVA; Figure 4(c)), similar to what had been observed in WT FoxP3^EGFP^ mice. Importantly, in* Tgfb1* ^−/−^ mice at any age, Treg frequency was no different in spleen versus liver (*P*: n.s.). Therefore, the transient increase in liver Treg frequency observed in the postnatal period requires TGF-*β*1.

### 3.3. Commensal Bacteria and MyD88 Are Required for the Increase in Liver Treg Frequency in the Early Postnatal Period

**Figure 5:**
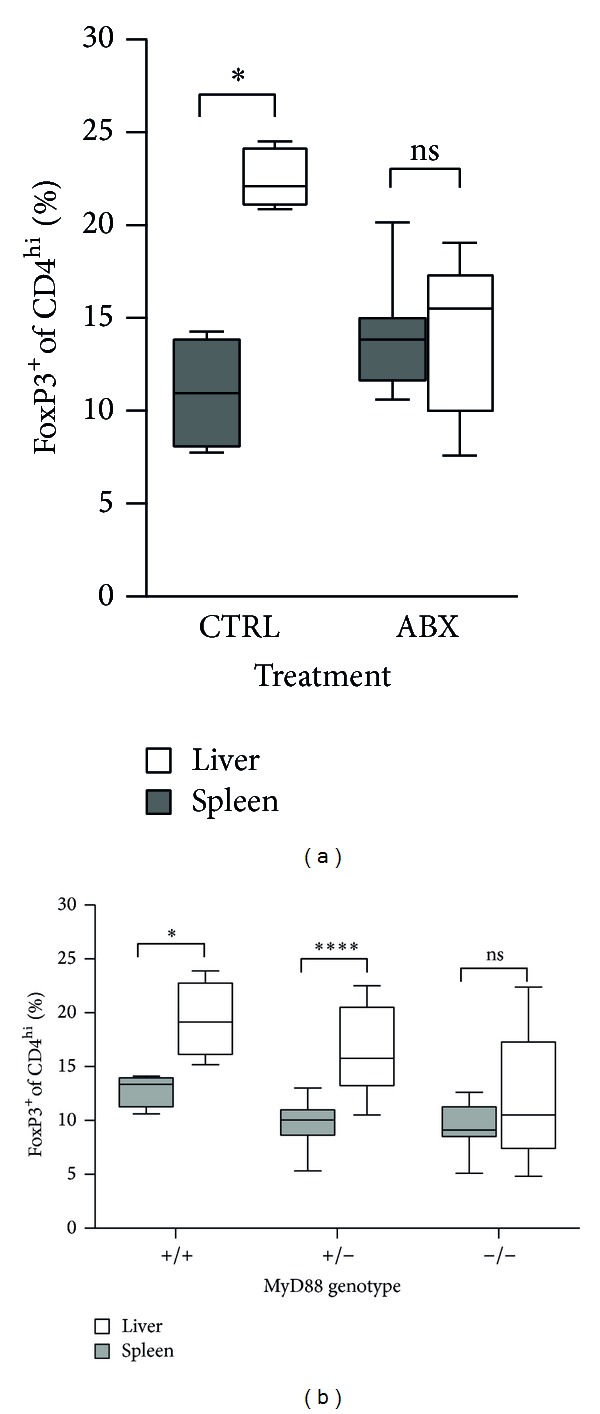
The increased frequency of FoxP3^+^CD4^+^ cells in postnatal liver is blocked by treatment with antibiotics and depends on MyD88. (a) Pregnant C57Bl/6 females were treated continuously with a cocktail of oral antibiotics from two weeks of gestation until pups were 8-9 days of age, at which time spleen and liver NPC were isolated and FoxP3^+^CD4^+^ cells were measured (*n* = 13 mice). Control mice are untreated C57Bl/6 background pups of the same age (*n* = 4 mice). Statistical analyses used the nonparametric Mann-Whitney test. (b) Liver and spleen FoxP3^+^CD4^+^ cell frequency data are shown for 8- to 9-day-old littermate* Myd88* ^+/+^ mice*, Myd88* ^+/−^ mice,* and Myd88* ^−/−^ mice. *N* = 8 to 13 mice per time point.* Myd88* mice were on a mixed BALB/c × C57Bl/6 background, but littermates were used, minimizing background effects. Statistical analyses used the nonparametric Mann-Whitney test.

We sought to further understand the factors that contribute to the transient increase in frequency of liver Tregs that occurs one week after parturition. We considered that the transient increase might represent a response to postnatal colonization of the murine intestine. Commensal organisms begin to colonize the murine intestinal tract as early as day 1 after birth [25]. The intestinal microbiota is known to play an important role in shaping the mature immune system in the intestine as well as extraintestinally [26]. Based on the anatomic relationship between the intestine and the liver, it is reasonable to conjecture that the gut-liver axis is important for the establishment of the immune system in the liver. We attempted to experimentally manipulate commensal colonization by treating pregnant dams with a cocktail of antibiotics before and after delivery. We obtained data from two litters in which the mothers had been treated successfully with oral antibiotics. Indeed, treatment with oral antibiotics abrogated the transient increase in liver Tregs (Figure 5(a)). In general, however, treatment with antibiotics resulted in unexpected and unacceptable morbidity and poor maternal behavior in mothers (not shown), so we took an orthogonal approach to test our hypothesis.

We hypothesized that the transient postnatal increase in liver Treg frequency is dependent on signals emanating from TLR responses to bacterial products. Since MyD88 is a common adaptor molecule that mediates downstream signaling from all bacterial responsive TLRs, we hypothesized that MyD88 is required for the transient postnatal increase in liver Treg frequency. We tested this hypothesis by examining Treg frequency in mice deficient in MyD88. We interbred heterozygous* Myd88* ^+/−^ mice to produce littermate* Myd88* ^+/+^ pups,* Myd88* ^+/−^ pups, and* Myd88* ^−/−^ pups. Importantly, a recent study shows that Myd88 gene status does not affect colonization of the intestine by commensal organisms [27]; therefore all mice from the same litter should become colonized with similar microbiota at similar concentrations, removing a potential artifact in interpretation of data. We analyzed mice at a time point (days 8-9) at which the difference in frequency between spleen Treg and liver Treg is expected to be maximal. Because these mice do not harbor the FoxP3^EGFP^ reporter construct, we directly analyzed intracellular FoxP3 expression on flow cytometry. As expected, wild type* Myd88* ^+/+^ pups had a higher frequency of Treg cells in liver than in spleen. Notably, the increase in liver Treg frequency at this age was completely abrogated in* Myd88* ^−/−^ pups, which had the same frequency of Treg cells in liver as in spleen (Figure 5(b)).* Myd88* ^+/−^ livers also showed a relative increase in liver Treg frequency, but this was more modest than in their wild type* Myd88* ^+/+^ littermates, suggesting there is a gene dosage effect in this pathway. Thus, MyD88 is required for the transient increase of Treg frequency seen in murine liver at 1-2 weeks of age, strongly supporting the hypothesis that the increase in Treg frequency in liver is a response to colonization of the gut by commensal organisms.

### 3.4. TLR2 Is Not Required for the Increase of Postnatal Liver Treg

**Figure 6:**
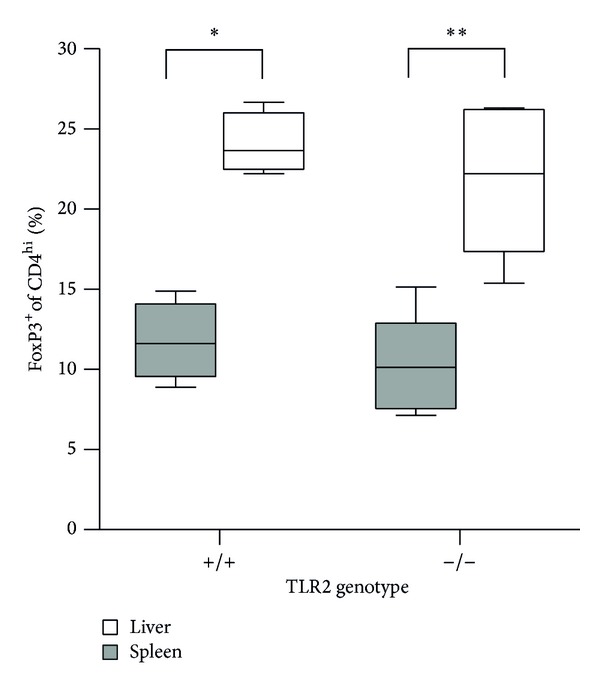
The increased frequency of FoxP3^+^CD4^+^ cells in postnatal liver is independent of TLR2. Liver and spleen FoxP3^+^CD4^+^ cell frequency data are shown for 8- to 9-day-old littermate C57Bl/6-background* Tlr2* ^+/+^ mice and* Tlr2* ^−/−^ mice. *N* = 4 to 5 mice per genotype. Statistical analyses used the nonparametric Mann-Whitney test.

As MyD88 is required for appropriate neonatal Treg development in liver, we next sought to identify upstream signaling molecule(s) that may be required for detection of gut colonization. TLR2 has been shown to be required for induction and upregulation of Tregs in mice [28, 29]. Moreover, TLR2 deficient mice exhibit a 50% decrease in the frequency of circulating Treg cells, whereas TLR4 deficient mice have normal Treg frequency [30, 31]. We therefore hypothesized that TLR2 is required for the transient increase of Treg frequency in murine liver during early development. We tested this hypothesis by examining Treg frequency in the liver and spleen of TLR2 deficient pups. As expected,* Tlr2* ^+/+^ mice had a higher frequency of Treg cells in liver than in spleen (analyzed at days 8-9).* Tlr2* ^−/−^ mice also had a higher frequency of Treg cells in liver than in spleen and were indistinguishable in this regard from their* Tlr2* ^+/+^ littermates (Figure 6). Thus, TLR2 is not required for the transient increase in Treg frequency. The involvement of MyD88 in this response may be downstream of a different TLR, or perhaps a combination of TLRs.

## 4. Discussion

While previous reports have studied the development of Tregs in the neonatal thymus [13], nothing is known about the development of this important T cell compartment in the neonatal liver. This study reveals that the development of the Treg compartment in the liver starts as early as day 3 after birth and that there is a pattern of a transient increase in the percentage of Tregs in the liver between days 6 and days 12. This pattern of development of postnatal liver Tregs requires TGF-*β* as well as MyD88. Colonization of the gut by commensal bacteria is seen as early as day 1 after birth [25]. It has been shown previously that the intestinal colonization of germ-free mice leads to the induction, activation, and expansion of mucosal Tregs [32] and the maintenance of immune homeostasis. Since the liver receives approximately 70% of its blood flow via the portal vein from the gut, the gut-liver axis plays a very important role in modulating the immune microenvironment of the liver.

Based on these previous observations and our findings here, we propose a model for liver Treg development. Microbial colonization of the intestine may be detected by intestinal cells by one or more TLR signaling pathways, mediated by the downstream adaptor molecule MyD88. The colonization of the gut also leads to the production of TGF-*β*1. Expression of TGF-*β* isoforms and receptors is detectable in rat gastric epithelium both during fetal development and in the neonatal stage [33]. In a model of induction of colonic Tregs with* Clostridium* species, it has been shown that, upon TLR ligation, intestinal epithelial cells increase TGF-*β*1 production and upregulate expression of matrix metalloproteinases (MMP2, MMP9, and MMP13) that hydrolyze latent TGF-*β*, converting it to its active form [34]. We propose that TLR/MyD88-mediated response to microbial colonization in the intestine enhances production of TGF-*β*1, resulting in an increase in Treg percentage in the liver. The observed transient increase in neonatal liver Tregs may result from expansion of Tregs within liver itself, or from an increased influx of Tregs from the intestine; the data presented here neither support nor exclude either possibility. TGF-*β*1 might act in the intestine to recruit naïve intestinal T cells along the Treg developmental pathway or cause expansion of an existing Treg population, which then traffic to the liver. Alternatively, microbial products resulting from gut colonization reaching the liver via the portal vein might only then be detected by TLRs expressed by resident liver cells. TLR expression in the liver is observed in several different cell populations, including Kupffer cells, hepatocytes, hepatic stellate cells, LSECs, hepatic dendritic cells, and liver NK cells [35, 36]. Interestingly, TLR4 ligation of quiescent HSCs results in increased expression of Bambi, the pseudoreceptor for TGF-*β*, increasing the sensitivity of HSCs to TGF-*β* [37].

TGF-*β*1 is critical for the development and maintenance of Treg cells in the thymus as well as in the periphery [4]. TGF-*β*1 is critical for the complete development of liver Tregs, since the increase in liver Tregs observed at one week postnatally is abrogated in mice deficient in the gene encoding TGF-*β*1. It has been shown that TGF-*β*1 may be acquired from the mother through breast-feeding [38] and pups here are indeed born from TGF-*β*1-replete mothers (*Tgfb1* heterozygous); moreover, there are two additional TGF-*β* isoforms expressed in mouse. Clearly, however, it is endogenously produced TGF-*β*1 that is critical for the Treg increase at one week after birth, and neither maternal TGF-*β*1 nor endogenous TGF-*β*2 or TGF-*β*3 is sufficient to rescue the liver Treg development phenotype.

In addition to TGF-*β*1, several additional secreted or membrane-bound factors are known to affect Treg development, including IL-2, retinoic acid and B-7 family molecules that signal through CD28 and CTLA-4 [39, 40]. It will be interesting to determine which, if any, of these factors participates in the regulation of liver Treg development and how they may interact with TGF-*β*1 and TLR/MyD88 to influence the liver Treg compartment.

Commensal bacteria play important roles in shaping the immune system [26, 41]. As previously noted, the presence or absence of an intact TLR/MyD88 response axis does not affect microbiota composition in steady state, and in fact maternal origin and vertical transmission are the important factors that define the structure of the microbiota in colonies of TLR knockout mice and MyD88 knockout mice [27]. In humans, it has been shown that maternal exposure to agriculture increases Tregs in cord blood, which might later affect allergic responses [42]. Our data using antibiotic treatment in wild-type mice, and the use of MyD88 deficient mice, suggest that commensals also contribute to shaping the composition of liver resident immune cells, specifically the Treg compartment. While MyD88 is a common adaptor molecule for all the bacterial responsive TLRs, it is also important in downstream signaling from IL-1R [43], and this pathway cannot be ruled out; however, the results of the antibiotics experiments argue that the relevant role of MyD88 is downstream of one or more TLRs. In our attempts to identify which TLR may be involved, we focused on TLR2 because of its defined role in Treg development [28, 29]. We found that TLR2 alone was dispensable for the Treg development pattern in the liver. The roles of other TLRs remain to be investigated.

Differential composition of the microbiota within the same genetic mouse strain, but obtained from different vendors, has been shown to result in differential development of the immune system [44, 45]. In this study we used mice bred in our animal facility, so we are not able to comment on whether differences in microbiota might differentially affect Treg development in the liver. We also do not know if the pattern of Treg development observed in liver is in response to one or more specific bacterial taxa, or if it represents a response to polymicrobial gut colonization.

It will be important to determine whether the transient increase in neonatal liver Treg frequency contributes to establishing proper liver immune cell function and if this increase in Treg frequency is important for the development of liver tolerance. A recent study using gene expression profiling has shown that exposure to microbiota in the neonatal period is essential for appropriate TLR responses, whose later exposure does not restore appropriately [41]. In this context, the timing of the increased liver Treg frequency may be important for establishing immune tolerance.

It is notable that the frequency of liver Tregs is very high one week after birth but then declines markedly; in the adult, the frequency of liver Tregs is much lower than the frequency of splenic Tregs. Presumably, as the frequency of liver Tregs declines, other tolerogenic pathways begin to have greater effect in maintaining the generally tolerogenic state of the liver. Such pathways likely include, as detailed in a comprehensive review by Crispe [5], expression of adhesion molecules to trap effector T cells in liver sinusoids, an abundance of immunosuppressive cytokines such as IL-10 and TGF-*β*, the expression of inhibitory T cell checkpoint molecules such as PD-L1, and the induction of apoptosis on T cells mediated by death ligands such as FasL that are expressed on various liver nonparenchymal cells, such as Kupffer cells.

## 5. Conclusion

In conclusion, our data suggest an important role for intestinal microbial colonization in the development of the liver Treg compartment. This might be important in the establishment of liver tolerance, and interruption or alteration of this physiologic event might contribute to inflammatory liver disease. In light of these studies, it is worthwhile to consider whether there may be a relationship between the use of antibiotics in the neonatal period and the subsequent development of inflammatory liver disease later in life.
